# Work from home or office during the COVID-19 pandemic: The different chain mediation models of perceived organizational support to the job performance

**DOI:** 10.3389/fpubh.2023.1139013

**Published:** 2023-03-03

**Authors:** Xiong Liu, Yumei Jing, Youyu Sheng

**Affiliations:** ^1^Faculty of Psychology, Tianjin Normal University, Tianjin, China; ^2^Mental Health Education and Counselling Centre, Hubei Normal University, Huangshi, China; ^3^Institute of Psychology, Chinese Academy of Sciences, Beijing, China

**Keywords:** perceived organizational support, job satisfaction, work engagement, job performance, work from home, COVID-19

## Abstract

With the coronavirus pandemic in 2019 (COVID-19), work from home (WFH) has become a frequent way of responding to outbreaks. Across two studies, we examined how perceived organizational support influences job performance when employees work in office or work from home. In study 1, we conducted a questionnaire survey of 162 employees who work in office. In study 2, we conducted a questionnaire survey of 180 employees who work from home. We found that perceived organizational support directly affected job performance when employees work in office. When employees work from home, perceived organizational support could not affect job performance directly. However, it could influence job performance indirectly through the separate mediating effects of job satisfaction and work engagement. These findings extend our understanding of the association of perceived organizational support and job performance and enlighten enterprises on improving employees' job performance during the COVID-19 pandemic.

## 1. Introduction

On March 11, 2020, the World Health Organization (WHO) declared the coronavirus disease 2019 (COVID-19) outbreak a pandemic; this pandemic has had major impacts around the world (1, 2). Forced closure of enterprises and industries around the world to curb the spread of the virus has brought a series of unique challenges to employees and employers (3). This change forced companies and employees to quickly adapt to work-from-home (WFH) policies (4). Gartner's survey of 229 HR departments revealed that about half of the companies had more than 80% of their employees working from home in the early stages of the COVID-19 pandemic, and estimated a large long-term increase in working from home after the pandemic (5).

Yet as many employees and employers had to suddenly work from home for the first time and without any preparation (6), and this sudden shift changed work arrangements and negatively impacted employees' physical and mental state as well as their work (2), which ultimately reduced job performance (7–9). WFH can lead to employees' loneliness, difficulties in team communication (10) and cooperation, and decreased job performance (11). For example, online communication lacks nonverbal cues, which increases loneliness (12) and is strongly negatively correlated with employees' affective commitment, affiliative behavior and job performance (13); online communication can lead to anxiety, confusion and communication errors among employees (14), and may even reduce the level of trust in teams (15). During a pandemic WFH prevent social connections and quality social interactions, which can also take a toll on employees' physical and mental health, further reducing job performance (16). Therefore, how to improve the job performance when the employees work from home is an urgent problem.

There are many important effects could improve the job performance, perceived organizational support, as a common variable in management and organizational behavior, is one of the most important ways for improving job performance (17–19). However, WFH has various negative impacts, such as increased loneliness, poor team communication and reduced trust, which may affect the mechanism of perceived organizational support.

Therefore, this study aims to compare how perceived organizational support influence employees' job performance when employees in WFH and work in office. In addition, many other factors might play a role in this mechanism, such as individual qualities, motivation, psychological capital, job satisfaction, we also put them into our considering model.

### 1.1. Relationship between perceived organizational support and job performance

Employees' job performance consists of a range of different activities that contribute to the organization in different ways; it is “employee behaviors that are relevant to the goals of the organization” (20). Job performance is also defined as the result of the function or indicator of a job or an occupation in a certain period of time (21).

Eisenberger proposed the concept of perceived organizational support, which referred to employee perceptions of how the organization views their contributions and cares about their interests. In short, perceived organizational support reflects the support employees feel from their organization (22). According to organizational support theory (OST), perceived organizational support is a valuable resource that will elicit norms of reciprocity in the process of social exchange, which will lead to greater employee efforts on behalf of the organization because of perceived indebtedness or perceived obligation and expected reward. Perceived organizational support also meets socioemotional needs, leading to greater identification and commitment to the organization, increased desire to help the organization succeed, and improved mental health (23–25). In addition, if an employee receives adequate training, resources, and support from their organization, he or she is more likely to expect the organization to achieve its goals and more likely to help the organization achieve its goals.

Some researchers have proposed that perceived organizational support can increase extra-role behaviors and reduce harmful behaviors to the organization; thus, they regard perceived organizational support as a predictor of job performance (17–19), which confirmed by several recent empirical studies (26–29). And perceived organizational support is positively correlated with job performance (30, 31), as demonstrated in previous studies. For example, a meta-analysis of 167 studies found that perceived organizational support has a moderate, positive effect on job performance (32). Shanock and Eisenberger (33) also found that perceived organizational support reduces behavior detrimental to the organization. Based on these studies, we believe that perceived organizational support has an undeniable positive impact on job performance.

However, the exact relationship between perceived organizational support and job performance remains controversial. Chen and Chen (34) discussed the degree of agreement between direct and indirect effects and empirical data, with results favoring a direct (rather than indirect) effect of perceived organizational support on job performance. However, recent research has suggested that perceived organizational support affects employees' job performance by generating positive emotions and gratitude based on social exchange processes (35). WFH due to the pandemic increased employee loneliness and socioemotional needs. Therefore, we believe that, for employees who work from home, the impact of perceived organizational support on job performance is more likely to occur through meeting employees' emotional needs, such as job satisfaction and work engagement. Based on these theories and empirical findings, we hypothesize the following: (a) In the condition of working in office, perceived organizational support directly affects job performance; (b1) In the condition of WFH, perceived organizational support indirectly affects job performance.

### 1.2. The relationships among job satisfaction, perceived organizational support, and job performance

Job satisfaction is a positive emotion that encompasses emotions such as joy, happiness, passion, enthusiasm and love (36). Others define job satisfaction as a positive emotional attitude toward work (37). Such positive emotions are generated when employees strongly feel that their organization cares for them and supports them. Meta-analyses and qualitative reviews of the literature on perceived organizational support have shown positive relationships between perceived organizational support and job satisfaction (17, 19, 24). This finding has been confirmed by recent empirical studies. A study with 127 school teachers found that perceived organizational support had a positive effect on both job and life satisfaction (38). A study of cement workers in Iran reached the same conclusion (39).

According to organizational support theory, when employees feel strongly supported by the organization, their socioemotional needs will be satisfied, which leads to increased job satisfaction. These employees will reciprocate by caring for the organization and doing their job well. A meta-analysis of 100 articles revealed a significant moderate positive relationship between job performance and job satisfaction (40). Dinc et al. (41) conducted a study on the job performance of nurses in hospitals and found that improvements in job satisfaction had a significant impact on nurse job performance. According to a study on 104 school principals and 313 teachers (42), a one-unit increase in job satisfaction of teachers led to a 10% increase in job performance. The support employees receive from the organization creates a positive impression and leads to positive results for both employees and the organization. Based on these findings, we hypothesize that (b2) in the condition of WFH, job satisfaction mediates the impact of perceived organizational support on job performance.

### 1.3. The mediating role of work engagement

Work engagement is defined as “a positive, fulfilling, work-related state of mind that is characterized by vigor, dedication, and absorption” (43) and is characterized by a high level of energy and strong identification with one's work (44).

According to the job demands-resources (JD-R) model, we suggest that perceived organizational support provides socioemotional support and is positively related to work engagement. When employees feel valued and supported by their organization, it enhances self-esteem and increases job satisfaction, thereby reinforcing their ability to manage work stress (45). Perceived organizational support also conveys the organization's evaluation of employee efforts and satisfies the employee's need for positive feedback and approval, which can also promote the intrinsic interest of employees and thus improve their work engagement (25). Other studies have also found that perceived organizational support is positively correlated with work engagement (46, 47).

In regard to the consequences of work engagement, numerous studies have linked work engagement to better health and positive emotions (48–52). Bakker (53) suggested that employees with higher levels of work engagement have higher job performance because (a) they experience positive emotions, which helps them to generate new ideas and resources, and (b) their health is improved, providing them with energy to work. Additionally, work engagement is regarded as a reasonable predictor of job performance because employees who most identify with their jobs tend to focus their thoughts on their jobs (54, 55). These findings have been empirically supported. Halbesleben and Wheeler (56) analyzed a sample of U.S. employees (*n* = 587), their supervisors, and their closest colleagues from a variety of industries and occupations and found that work engagement predicted not only higher self-reported in-role performance 2 months later but also higher in-role performance as rated by superiors and peers. Tisu et al. (57) analyzed a sample of Romanian workers and found that work engagement has positive effects on mental health and job performance. Based on these findings, we hypothesize that (b3) in the condition of WFH, work engagement mediates the impact of perceived organizational support on job performance.

### 1.4. The chain mediating effect of job satisfaction and work engagement

According to social exchange, employees and their organization form a positive emotional connection after a long-term successful exchange relationship and employees are more willing to improve the performance of the organization and make their own efforts to maintain such a social exchange relationship (58). While material reciprocity leads to temporary pleasure, spiritual reciprocity can bring long-term benefits. Organizational support includes not only material support but also spiritual support, such as attention, concern, encouragement and respect for employees. Recent research suggests that gratitude or other positive emotions generated by perceived organizational support may also help improve employees' job performance based on social exchange processes (35).

When perceived organizational support is high, employees are (under certain conditions) more likely to exhibit higher job performance and reduced absenteeism. However, some studies have shown different results. Stamper and Johlke (59) reported that perceived organizational support was not related to salespeople's task performance. In addition, some studies have suggested that perceived organizational support mediates multiple types of organizational experience variables and thus may not directly affect job performance (19, 30, 59).

An empirical study of 744 police officers in China found a nonsignificant direct effect of perceived organizational support on work engagement, but a significant indirect relationship of these variables mediated by job satisfaction (60). We discussed the mediating effects of job satisfaction and work engagement in the above section. According to social exchange theory, perceived organizational support, job satisfaction and work engagement meet the needs of employees; thus, these factors affect employees' job performance. Based on these findings, we hypothesize that (b4) in the condition of WFH, job satisfaction and work engagement exert a chain mediating effect on the relationship between perceived organizational support and job performance.

### 1.5. Overall hypothetical model

In conclusion, the research hypotheses were as follows:

(a) In the condition of working in office, perceived organizational support directly affects job performance;
(b1) In the condition of WFH, perceived organizational support indirectly affects job performance;
(b2) In the condition of WFH, job satisfaction mediates the impact of perceived organizational support on job performance;
(b3) In the condition of WFH, work engagement mediates the impact of perceived organizational support on job performance;
(b4) In the condition of WFH, job satisfaction and work engagement exert a chain mediating effect on the relationship between perceived organizational support and job performance.

To test these hypotheses, this study used two questionnaire surveys (one is in employees of working in office, one is in employees of WFH) to compare the different models of perceived organizational support and job performance.

## 2. Study 1

### 2.1. Subjects

This study was conducted online through a survey website. The survey website sent the link to the questionnaire to the email address of full-time employees who work in office, and the completed questionnaire was collected through the survey website. In this study, a screening question was included in the questionnaire to identify and exclude participants who did not answer carefully. One hundred sixty-two valid questionnaires were returned, with an effective recovery rate of 93.10%. All participants signed informed consent prior to filling out the questionnaire. They were paid 10 yuan for participating after completing the questionnaire. Among the participants, 67 were male (41.4%), and 95 were female (58.6%). The study was reviewed and approved by Ethics Committee of Hubei Normal University. All participants signed informed consent prior to filling out the questionnaire.

### 2.2. Materials

#### 2.2.1. Perceived Organizational Support Scale

This study used the Perceived Organizational Support Scale (POSS) developed by Ling et al. (61). The scale consists of 24 items scored on a 5-point Likert scale and is divided into three dimensions: work support, value identification and interest concern. The higher the total POSS score is, the better the respondent's perceived organizational support. The POSS demonstrates high reliability and suitable for the Chinese population. In this study, the internal consistency coefficient was 0.934.

#### 2.2.2. Minnesota Satisfaction Questionnaire

The Minnesota Satisfaction Questionnaire (MSQ) developed by Weiss et al. (62) was used to measure job satisfaction. The MSQ consists of 20 items with responses given on a 5-point Likert scale ranging from 1 (strongly disagree) to 5 (strongly agree). The higher the total score is, the higher the respondent's job satisfaction. The internal consistency coefficient for this study was 0.914.

#### 2.2.3. Utrecht Work Engagement Scale-9

The Utrecht Work Engagement Scale-9 (UWES-9) developed by Schaufeli et al. (63) is widely used; it was later revised by Zhang and Gan (64) to accommodate the cultural background of China. The UWES-9 consists of nine items scored on a 5-point Likert scale ranging from 1 (strongly disagree) to 5 (strongly agree). The higher the total score is, the higher the respondent's work engagement. The internal consistency coefficient for this study was 0.899.

#### 2.2.4. Job Performance Scale

The job performance scale (JPS) developed by Li et al. (65) was used in this study. This scale contains two dimensions: task performance and relationship performance, with a total of nine items. Among them, task performance is evaluated with five items, such as “I rarely make mistakes when completing work.” Relationship performance is evaluated with four items, such as “I treat my colleagues fairly” and “I offer to help my colleagues.” The nine items are scored on a 5-point Likert scale ranging from 1 (strongly disagree) to 5 (strongly agree). The higher the total score is, the higher the respondent's job performance. The internal consistency coefficient for this study was 0.763.

### 2.3. Statistical analysis

SPSS Statistic 26.0 (IBM SPSS Statistics, New York, United States) was used to perform general descriptive statistics and Pearson correlation analysis (two-sided *p* < 0.05 was considered significant). To ensure the accuracy of the results, the variance inflation factor (VIF) method was used to assess collinearity (VIF > 10 indicates serious collinearity between the variables, and the corresponding variables should be eliminated). Model 6 in the process plug-in compiled by Hayes (66) was used for chain mediating effect analysis, and the bias-corrected percentile bootstrap method was used to evaluate the significance of the mediating effect. If the 99% confidence interval (CI) did not contain 0, the effect was considered statistically significant (67). In addition, Harman's one-factor test was used to test for common method bias before analyzing the data (68).

### 2.4. Results

#### 2.4.1. Common method bias test

Because this study used self-report scales to collect data, which can lead to common method bias, the Harman single-factor method of exploratory factor analysis including perceived organizational support, job satisfaction, work engagement, and job performance was conducted. Only 34.847% of the variance was explained by the largest factor, which is less than the critical value of 40%, indicating that there was no significant common method bias in this study.

#### 2.4.2. Correlations among perceived organizational support, job satisfaction, work engagement, and job performance

Table 1 presents the means (*M*), standard deviations (SD) and correlations. The highest mean is job performance (4.265). Pearson's product-moment correlation analysis was used to analyze relationships among perceived organizational support, job satisfaction, work engagement, and job performance (see Table 1). The results showed that ① perceived organizational support was significantly positively correlated with job satisfaction, work engagement and job performance (*r* = 0.871, *p* < 0.01; *r* = 0.806, *p* < 0.01; and *r* = 0.638, *p* < 0.01, respectively); ② work engagement was significantly positively correlated with job performance and job satisfaction (*r* = 0.573, *p* < 0.01 and *r* = 0.774, *p* < 0.01, respectively); and ③ job satisfaction was significantly positively correlated with job performance (*r* = 0.593, *p* < 0.01).

**Table 1:** Descriptive statistics and correlation matrix for each variable.

	***M***	**SD**	**Perceived organizational support**	**Job satisfaction**	**Work engagement**	**Job performance**
Perceived organizational support	3.619	0.651	1			
Job satisfaction	3.810	0.609	0.871^**^	1		
Work engagement	3.583	0.771	0.806^**^	0.774^**^	1	
Job performance^*^	4.265	0.378	0.638^**^	0.593^**^	0.573^**^	1

* *p* < 0.05.

** *p* < 0.01.

#### 2.4.3. Relationship between perceived organizational support and job performance: A chain mediation model

The above analysis showed significant correlations among the variables and the presence of possible collinearity.

**Figure 1:**
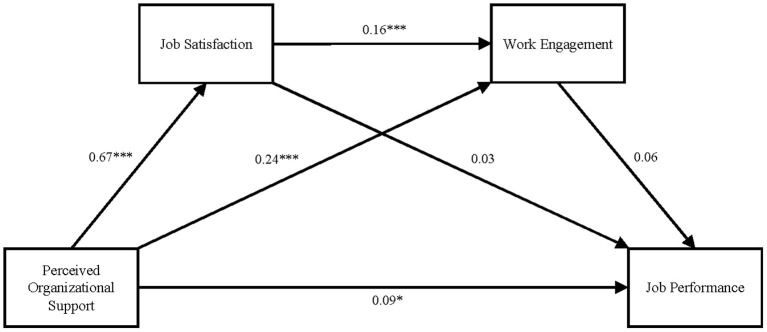
The chain mediation model of working in office. *p < 0.05 and ***p < 0.001.

Therefore, before testing the chain mediating effect, the predictive variables in the equation were standardized, and collinearity diagnostics were performed. The results showed that the VIF values (5.050, 4.419, and 3.038) of all of the predictors were <10. Therefore, there was no serious collinearity in the data used for this study, indicating that these data were suitable for further mediation analysis (see Figure 1).

The process plug-in developed by Hayes was used to evaluate the 95% CI of the mediating effects of job satisfaction and work engagement on the relationship between perceived organizational support and job performance (the bootstrap sample size was 5,000). The results showed that perceived organizational support significantly positively predicted job performance, job satisfaction and work engagement (β = 0.09, *p* < 0.05; β = 0.67, *p* < 0.001; and β = 0.24, *p* < 0.001, respectively); that job satisfaction significantly predicted work engagement (β = 0.16, *p* < 0.001) but did not significantly predict job performance (β = 0.03, *p* > 0.05); and that work engagement did not significantly predict job performance (β = 0.06, *p* > 0.05).

Further testing of the mediating effect showed that the bootstrap 95% CI of the total indirect effect of job satisfaction and work engagement on the relationship between perceived organizational support and job performance was −0.0323 to 0.1134. This interval included 0, indicating that the chain mediating effect of job satisfaction and work engagement on the relationship between perceived organizational support and job performance was not significant. Thus, a chain mediation model was not established and Hypothesis (a) was supported.

## 3. Study 2

### 3.1. Subjects

Study 2 adopted the same online survey method as Study 1 and took place during the same period. However, unlike those in Study 1, the participants in Study 2 were full-time employees who work from home. A total of 189 questionnaires were distributed, and 180 valid questionnaires were returned, for an effective recovery rate of 95.23%. All participants signed informed consent forms prior to filling out the questionnaire. Participants were paid 10 yuan after completing the questionnaire. The study was reviewed and approved by Ethics Committee of Hubei Normal University. All participants signed informed consent prior to filling out the questionnaire.

### 3.2. Materials

Study 2 adopted the same four questionnaires as Study 1: the POSS, MSQ, UWES-9, and JPS.

### 3.3. Statistical analysis

Study 2 used the same statistical analysis approach as Study 1.

### 3.4. Results

#### 3.4.1. Common method bias test

With the Harman single-factor method, perceived organizational support, job satisfaction, work engagement, and job performance were included in an exploratory factor analysis. Only 35.140% of the variance was explained by the largest factor, which was less than the critical value of 40%, indicating that there was no significant common method bias in this study.

#### 3.4.2. Correlations among perceived organizational support, job satisfaction, work engagement, and job performance

Table 2 presents the means (*M*), standard deviations (SD) and correlations. The highest mean is job performance (4.215). Pearson's product-moment correlation analysis was used to analyze perceived organizational support, job satisfaction, work engagement, and job performance (see Table 2). The results showed that ① perceived organizational support was significantly positively correlated with job satisfaction, work engagement and job performance (*r* = 0.806, *p* < 0.01; *r* = 0.674, *p* < 0.01; and *r* = 0.552, *p* < 0.01, respectively); ② work engagement was significantly positively correlated with job performance and job satisfaction (*r* = 0.549, *p* < 0.01 and *r* = 0.751, *p* < 0.01, respectively); and ③ job satisfaction was significantly positively correlated with job performance (*r* = 0.605, *p* < 0.01).

**Table 2:** Descriptive statistics and correlation matrix for each variable.

	***M***	**SD**	**Perceived organizational support**	**Job satisfaction**	**Work engagement**	**Job performance**
Perceived organizational support	3.719	0.665	1			
Job satisfaction	3.899	0.574	0.806^**^	1		
Work engagement	3.621	0.791	0.674^**^	0.751^**^	1	
Job performance^*^	4.215	0.400	0.552^**^	0.605^**^	0.549^**^	1

* *p* < 0.05.

** *p* < 0.01.

#### 3.4.3. Relationship between perceived organizational support and job performance: A chain mediation model

The above analysis showed that there were significant correlations among the variables and the presence of possible collinearity. Therefore, before testing the chain mediating effect, the predictive variables in the equation were standardized, and collinearity diagnostics were performed. The results showed that the VIF values (2.949, 3.688, and 2.364) of all of the predictors were <10. Therefore, there was no serious collinearity in the data used for this study, indicating that these data were suitable for further mediation analysis.

**Figure 2:**
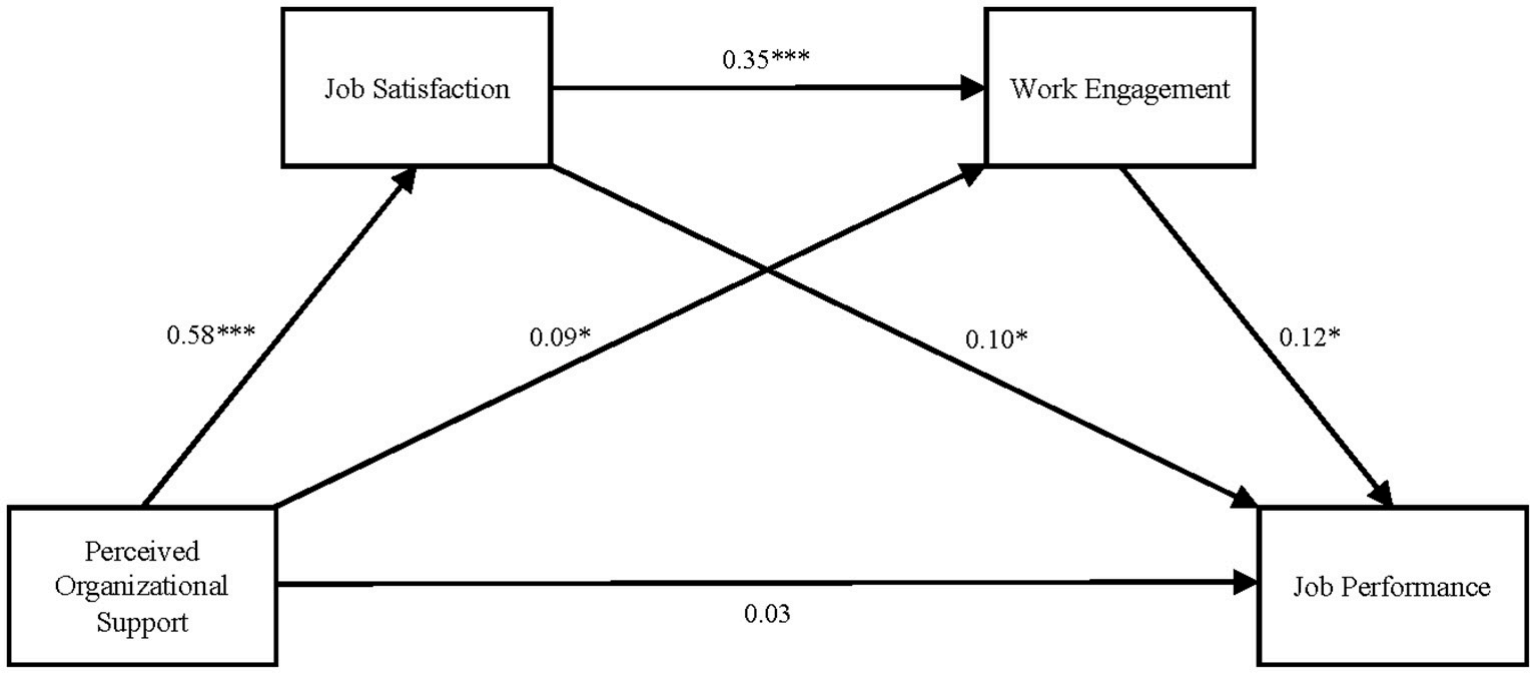
The chain mediation model of WFH. *p < 0.05 and ***p < 0.001.

The process plug-in developed by Hayes was used to evaluate the 95% CI of the chain mediating effect of job satisfaction and work engagement on the relationship between perceived organizational support and job performance (the bootstrap sample size was 5,000), and a chain mediation model was established (see Figure 2). The results showed that perceived organizational support significantly positively predicted job satisfaction and work engagement (β = 0.58, *p* < 0.001 and β = 0.09, *p* < 0.05, respectively) but did not significantly predict job performance (β = 0.03, *p* > 0.05); that job satisfaction significantly predicted work engagement and job performance (β = 0.35, *p* < 0.001 and β = 0.10, *p* < 0.05, respectively); and that work engagement significantly predicted job performance (β = 0.12, *p* < 0.05).

Further mediation analysis (see Table 3) showed that the bootstrap 95% CI of the total effect of job satisfaction and work engagement on the relationship between perceived organizational support and job performance was 0.0975–0.1538. This interval did not include 0; thus, job satisfaction and work engagement mediated the relationship of perceived organizational support and job performance. These two factors had a total indirect effect of 0.095, accounting for 75.18% of the total effect. This mediating effect was mainly composed of the following three paths: (1) perceived organizational support → job satisfaction → job performance [95% CI = (0.0072, 0.1111), standard error (SE) = 0.0262], under which the mediating effect is 0.0604, accounting for 48.05% of the total effect, and Hypothesis (b2) was supported; (2) perceived organizational support → work engagement → job performance [95% CI = (0.0006, 0.0297), standard error (SE) = 0.0076], under which the mediating effect was 0.0106, accounting for 8.43% of the total effect, and Hypothesis (b3) was supported; and (3) perceived organizational support → job satisfaction → work engagement → job performance [95% CI = (0.0035, 0.0470), standard error (SE) = 0.0108], under which the mediating effect was 0.0235, accounting for 18.70% of the total effect, and Hypotheses (b1) and (b4) were supported.

**Table 3:** Bootstrap analysis of the mediation analysis.

	**Effect**	**Bootstrap SE**	**Bootstrap CI**	**Bootstrap CI**
Indirect effect 1	0.0604	0.0262	0.0072	0.1111
Indirect effect 2	0.0106	0.0076	0.0006	0.0297
Indirect effect 3	0.0235	0.0108	0.0035	0.047

## 4. Discussion

This study explored the effect of perceived organizational support on job performance and the mediating effects of job satisfaction and work engagement. The results indicated that WFH influenced the relationship between perceived organizational support and job performance. In the condition of working in office, perceived organizational support directly affected job performance. In the condition of WFH, perceived organizational support indirectly affected job performance. In addition, in the condition of WFH, our results confirmed the separate mediating effects of job satisfaction and work engagement; moreover, job satisfaction and work engagement exerted a chain mediating effect on the relationship between perceived organizational support and job performance.

In our study, perceived organizational support was significantly positively correlated with organizational behavioral variables such as job performance, job satisfaction and work engagement, similar to previous research results. Thus, perceived organizational support is an important psychological variable that merits special attention in research and work applications.

Additionally, WFH influenced the relationship between perceived organizational support and job performance. The mechanism of action by which perceived organizational support influences job performance is controversial. Some researchers believe that perceived organizational support mainly affects job performance in a direct manner (30, 69). Other researchers believe that perceived organizational support influences job performance mainly through mediating factors such as job satisfaction, positive affectivity, affective commitment, organization-based self-esteem and organizational citizenship behavior (70–72). Different from previous research results, the conclusion of this study shows that the effect path of perceived organizational support on job performance is not fixed, which is affected by work mode. This study enriches the gap of research on WFH.

Regardless of this debate, perceived organizational support has a significant impact on job performance according to the principle of reciprocity. We believe that employees who work in office tend to regard organizational support as beneficial for organization. Based on the principle of reciprocity, when employees feel supported by their organization, they will be willing to make efforts to repay the organization for this perceived support, such as by improving job performance and increasing organizational citizenship behaviors. This exchange is more straightforward. Chen and Chen (34) uses affective support and instrumental support to explore the impact of perceived organizational support on job performance, and draws the conclusion that the direct effect is greater than the indirect effect, which is consistent with the conclusion of this study. In the condition of working in office, the direct effect of perceived organizational support on job performance is greater than in the condition of WFH.

However, employees believed that the organizational support experienced while working from home was more real than that experienced during working in office. On the one hand, employees who work from home are unable to communicate with their supervisors or colleagues in an informal and face-to-face manner due to their separate work location; thus, they usually rely on regular formal online meetings to exchange and share information and opinions. However, online communication can lead to information loss and low communicative efficiency (12). The above factors make it difficult for employees who work from home to achieve high-quality communication and objective exchanges, which may explain why there was a greater indirect effect of perceived organizational support on job performance than the direct effect. On the other hand, due to the lack of daily face-to-face interaction and communication with supervisors and colleagues, employees who work from home may experience social isolation or even envy of their colleagues (73, 74). In addition to limiting the freedom of movement, COVID-19 lockdowns are also associated with a variety of emotional challenges, including concrete fears of infection, frustration, and anger, as well as more generalized and severe symptoms of anxiety, depression, and posttraumatic stress (75, 76). The research of Armeli et al. (77) on 308 police patrolmen showed a nonsignificant correlation between perceived organizational support and job performance in subjects with weak socioemotional needs, which indicates that positive emotions may affect the relationship between perceived organizational support and job performance. Zhou and Bao (78) measured the perceived organizational support and only investigated the affective support, and concluded that the impact of perceived organizational support on job performance is mostly through indirect effects. Therefore, we believe that in the context of COVID-19, employees who work from home have greater emotional needs that can be met by perceived organizational support to increase job satisfaction and work engagement, thereby indirectly improving job performance.

During the COVID-19 pandemic, WFH is an effective governmental implementation to prevent further spread of disease; however, WFH impact both organizations and employees. It is urgent to identify ways to maintain job performance of employees who work from home. According to our results, perceived organizational support positively impacts employees' job performance in different work scenarios. This study explored the possible factors influencing job performance and validates and extends previous findings. Additionally, this study provides insight into the mechanism by which job satisfaction and work engagement influence the relationship between perceived organizational support and job performance and provides a possible direction for future to improve employees' job performance.

However, the current study has some limitations. First, its cross-sectional nature prevents. However us from drawing any conclusions about causal relationships. ThereforeHowever, the direction of relationships among perceived organizational support, job satisfaction, work engagement, and job performance cannot be determined. Future longitudinal studies should use cross-lagged analysis to examine bidirectional associations among these variables. Second, only two mediating variables, job satisfaction and work engagement, were examined in the present study. Future researchers should consider more mediating mechanisms, such as stress, anxiety, and leadership style, that influence the relationship between perceived organizational support and job performance. Finally, due to the limitations of data collection during a pandemic, all studied variables were derived from the same source. The scope and sources of data collection should be expanded in future studies.

## 5. Conclusions

In the condition of working in office, perceived organizational support directly affected job performance. In the condition of WFH, perceived organizational support indirectly affected job performance. And perceived organizational support affected job performance through the separate mediating effects of job satisfaction and work engagement. Additionally, in this condition, perceived organizational support affected job performance through the chain mediating effect of job satisfaction and work engagement. These findings extend our understanding of the association of perceived organizational support and job performance and enlighten enterprises on improving employees' job performance during the COVID-19 pandemic.

## Data availability statement

The raw data supporting the conclusions of this article will be made available by the authors, without undue reservation.

## Ethics statement

The studies involving human participants were reviewed and approved by Hubei Normal University. The patients/participants provided their written informed consent to participate in this study.

## Author contributions

Conceptualization and writing—original draft: XL and YS. Data curation: XL. Funding acquisition and methodology: YJ. Project administration, supervision, and validation: YS. Writing—review and editing: XL, YJ, and YS. All authors contributed to the article and approved the submitted version.
